# Integrated Genome Mining, Bacterial Co-Culture Activation, and Peptidomic Analyses Identify Antimicrobial Peptide Candidates from South American Bacteria

**DOI:** 10.3390/antibiotics15070696

**Published:** 2026-07-16

**Authors:** Abraham Espinoza-Culupú, Samantha Rubio Vasquez, Irving Vílchez Toribio, Mariella Farfán-López, Brizeth Molina Ramos, Mario Cueva Távara, Ana Paula Palacios-Rodriguez, Pedro Ismael da Silva Junior, Pablo Ramirez

**Affiliations:** 1 Laboratory of Molecular Microbiology and Biotechnology, Faculty of Biological Sciences, Universidad Nacional Mayor de San Marcos, Lima 15081, Peru; aespinozac@unmsm.edu.pe (A.E.-C.); samantha.rubio@unmsm.edu.pe (S.R.V.); irving.vilchez@unmsm.edu.pe (I.V.T.); bmelissa.molina@outlook.com (B.M.R.); mcuevata@unmsm.edu.pe (M.C.T.); 2 Laboratory of Environmental Microbiology and Biotechnology, Faculty of Biological Sciences, Universidad Nacional Mayor de San Marcos, Lima 15081, Peru; mariella.farfan@unmsm.edu.pe; 3 Butantan Institute, Laboratory for Applied Toxinology, Center of Toxins, Immune-Response and Cell Signalling—CeT-ICS/CEPID, São Paulo 05503-900, Brazil; ana.palacios@usp.br (A.P.P.-R.);; 4 Post-Graduation Program Interunits in Biotechnology, USP/IBu/IPT—Institute of Biomedical Sciences, University of São Paulo, São Paulo 05508-900, Brazil

**Keywords:** genome and metagenome mining, bacterial co-culture, bioactive secretomes, peptidomics, antimicrobial peptides

## Abstract

**Background/Objectives**: Antimicrobial resistance (AMR) is a major global health threat that requires the discovery of new antimicrobial agents. Environmental microbiomes from understudied regions represent a valuable source of antimicrobial peptide (AMP) candidates. This study aimed to identify and prioritize AMP candidates from South American genomic and metagenomic datasets and to investigate the antimicrobial potential of bioactive secretomes obtained through bacterial co-culture. **Methods**: A total of 853 genomes and 360 metagenomes were analyzed using a reproducible genome- and metagenome-mining pipeline combined with machine learning-based AMP prediction. Predicted AMP candidates were further characterized using complementary bioinformatic tools to assess physicochemical, structural, hemolytic, toxicological, anti-inflammatory, and anticancer properties. Selected environmental isolates were subjected to bacterial co-culture, followed by SPE-C18 and HPLC fractionation. Antimicrobial activity, antioxidant activity, hemolysis, minimum inhibitory concentration (MIC), and LC-MS/MS peptidomic analyses were performed on bioactive secretome fractions. **Results**: Genome and metagenome mining identified diverse AMP candidate sequences associated with bacterial genera including *Streptomyces*, *Bacillus*, *Burkholderia*, and *Shewanella*. Structural predictions revealed a predominance of α-helical conformations among prioritized candidates. Several secretome fractions obtained from co-cultures displayed antimicrobial activity against Gram-positive and Gram-negative bacteria, including methicillin-resistant *Staphylococcus aureus* (MRSA). Active fractions showed no detectable hemolytic activity and exhibited antioxidant activity in DPPH assays. MIC analyses indicated broad-spectrum activity against *Escherichia coli* ATCC 11229, *Pseudomonas aeruginosa* ATCC 27853, *Klebsiella pneumoniae*, carbapenem-resistant *Acinetobacter baumannii*, and MRSA, with an apparent MIC of 10,000 mg/L. LC-MS/MS analysis of bioactive fractions identified peptide sequences by de novo sequencing, including KTESHHK, KRVGPRR, GLFPRLGVSPR, and HHAEHLVHFR. **Conclusions**: Integrated genome mining, bacterial co-culture activation, and peptidomic analyses provide a useful framework for prioritizing antimicrobial peptide candidates from environmental microbiomes. The identification of peptide-containing bioactive fractions with antimicrobial and antioxidant activities highlights the potential of South American bacterial resources for the discovery of novel antimicrobial compounds. Further purification, peptide synthesis, and biological validation will be required to determine the contribution of individual peptides to the observed activities.

## 1. Introduction

The rapid emergence and dissemination of antimicrobial resistance (AMR) represent one of the most pressing challenges to global public health. The effectiveness of many conventional antibiotics has progressively declined due to the increasing prevalence of multidrug-resistant pathogens, limiting available therapeutic options and increasing the burden of infectious diseases [1,2,3,4]. Current projections estimate that AMR could be responsible for up to 10 million deaths annually by 2050 if effective interventions are not implemented [3]. Consequently, the discovery of novel antimicrobial agents has become a major priority in both clinical and environmental microbiology.

Antimicrobial peptides (AMPs) have attracted considerable attention as promising alternatives to conventional antibiotics. These molecules are naturally produced by a wide range of organisms and constitute an important component of innate defense systems [5,6]. In addition to their broad-spectrum antimicrobial activity, several AMPs exhibit immunomodulatory [7], anti-inflammatory [8], antioxidant [9], and anticancer properties [10], highlighting their potential for therapeutic development [11]. Their diverse mechanisms of action, particularly membrane disruption, may also reduce the likelihood of resistance development compared with traditional antibiotics [12].

Recent advances in genome and metagenome mining have created new opportunities for the discovery of AMP candidates from environmental microorganisms. The increasing availability of sequencing data, together with machine learning-based prediction tools, enables large-scale screening of genomic resources and facilitates the identification of peptides with favorable antimicrobial and physicochemical characteristics [13,14,15,16]. Several studies have demonstrated the value of combining computational prediction with environmental microbiology approaches to identify promising antimicrobial candidates from previously unexplored microbial communities [17,18].

In parallel, bacterial co-culture has emerged as an effective strategy for stimulating the production of bioactive metabolites that are not expressed under standard laboratory conditions. Microbial interactions can activate silent biosynthetic pathways and trigger competitive responses, increasing the probability of detecting compounds with antimicrobial activity [19,20].

In this study, we combined genome and metagenome mining, machine learning-based prediction, bacterial co-culture activation, and peptidomic analyses to identify and prioritize antimicrobial peptide candidates from South American bacterial resources. In addition, bioactive secretomes obtained from selected environmental isolates were evaluated for antimicrobial, antioxidant, and hemolytic activities, and active fractions were further characterized by LC-MS/MS. This integrated approach was used to explore the potential of South American environmental bacteria as a source of novel antimicrobial compounds.

## 2. Results

### 2.1. Overview of the Genomic and Metagenomic Datasets Analyzed

A total of 853 bacterial genomes and 360 metagenomic datasets retrieved from South American environments were analyzed using the genome and metagenome mining pipeline for antimicrobial peptide discovery. These datasets provided the basis for the identification and prioritization of candidate antimicrobial peptides, which are described in the following sections. Detailed information on the analyzed datasets, including country of origin, SRA accession numbers, sequencing metadata, library preparation strategies, sequencing platforms, and library layouts, is provided in Supplementary Tables S1 and S2.

### 2.2. Distribution of Genomic and Metagenomic Sources Associated with AMP Candidates

A large-scale screening of South American genomic and metagenomic datasets was conducted to identify candidate antimicrobial peptides (AMPs). Application of the Macrel prediction pipeline using an AMP probability threshold ≥ 0.6 resulted in the identification of 37 genome-derived and 26 metagenome-derived peptide candidates. These sequences were subsequently evaluated for predicted antimicrobial, hemolytic, toxicological, anti-inflammatory, anticancer, physicochemical, and structural properties.

The bacterial genera represented in the genomic datasets and the environmental sources included in the metagenomic datasets are summarized in Supplementary Figures S1 and S2, respectively. The identified AMP candidates were distributed across multiple bacterial genera and environmental sources from South American ecosystems, expanding the repertoire of predicted antimicrobial peptides identified by the genome and metagenome mining pipeline.

### 2.3. Functional Characterization of Genome and Metagenome-Derived AMP Candidates

Supplementary Tables S3 and S4 summarize the peptide sequences identified through the genome and metagenome mining pipeline. A total of 37 genome-derived and 26 metagenome-derived peptide candidates were further characterized using complementary prediction tools to evaluate their antimicrobial activity, hemolytic behavior, toxicity, physicochemical characteristics, and additional biological properties. Based on the combined prediction results, eight genome-derived and four metagenome-derived peptide candidates fulfilled all prioritization criteria, including antimicrobial activity predicted by both AMP Scanner v2 and CAMPR3, non-hemolytic behavior predicted by Macrel and HemoPI2.0, non-toxicity predicted by ToxinPred3, and anti-inflammatory activity predicted by PreAIP. Representative candidates included the genome-derived peptide GLGFKGLGFCDSRFYGIGFRV (SRR30832042) and the metagenome-derived peptide AVEGLSKRFPKLAGKP (SRR14932608c) (Supplementary Tables S3 and S4). These peptide candidates were considered the most promising for future experimental validation.

In addition to their predicted antimicrobial activity, several candidates were also predicted to exhibit anti-inflammatory and anticancer properties according to PreAIP, PepNet, antiCP 2.0, and mACPpred analyses (Supplementary Tables S3 and S4). These predicted activities were not experimentally evaluated in the present study and should therefore be considered preliminary computational predictions that require further biological validation.

### 2.4. Structural Prediction of Prioritized AMP Candidates

A subset of AMP candidates was selected for structural modeling based on their predicted antimicrobial activity, non-hemolytic behavior, low toxicity, and favorable physicochemical properties. Three dimensional structural predictions were generated using AlphaFold3 to further explore the structural features of the prioritized candidates.

**Figure 1:**
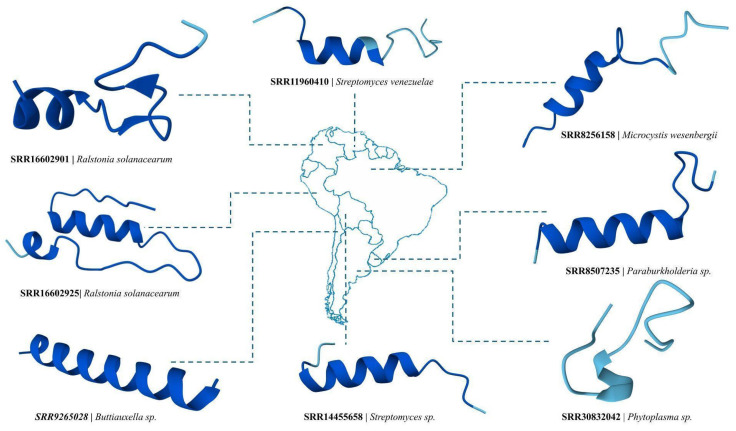
Predicted three-dimensional structures of prioritized AMP candidates identified from South American bacterial genomes. Structural models were generated using AlphaFold3 for peptide candidates selected based on predicted antimicrobial activity, non-hemolytic behavior, and low toxicity. Each model is accompanied by its corresponding SRA accession number, geographic origin, and bacterial genus.

**Figure 2:**
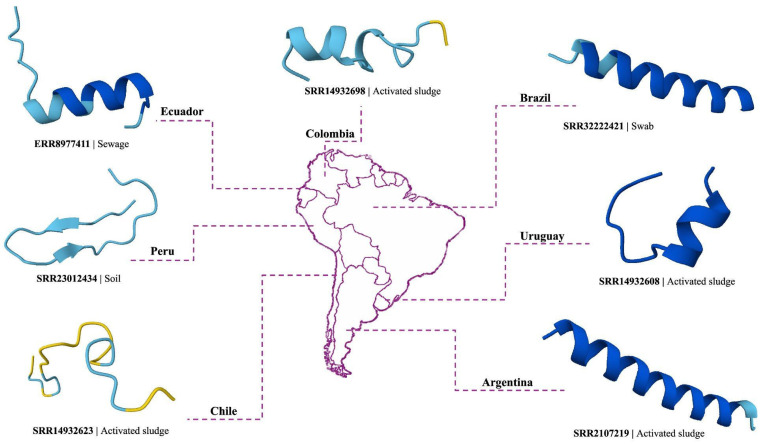
Predicted three-dimensional structures of prioritized AMP candidates identified from South American metagenomic datasets. The displayed models correspond to peptide candidates selected through the integrated computational screening pipeline. The SRA accession number and environmental source associated with each metagenomic dataset are indicated. Most predicted structures exhibited α-helical motifs with flexible terminal regions.

The predicted models revealed a predominance of α-helical conformations among the prioritized peptides candidates. Peptides derived from genomic datasets (Figure 1) exhibited moderate structural diversity, including short α-helices and flexible regions, whereas candidates identified from metagenomic datasets (Figure 2) displayed more defined helical motifs. α-Helical structures are frequently reported among antimicrobial peptides because they facilitate interactions with bacterial membranes, supporting the predicted antimicrobial potential of these candidates. Nevertheless, these structural features are based exclusively on computational predictions and require experimental validation.

### 2.5. Co-Culture and Pre-Fractionation of Bioactive Secretomes

Genome and metagenome mining analyses highlighted bacterial genera frequently associated with predicted AMP candidates, including members of the genera *Shewanella* and *Streptomyces*. Environmental isolates belonging to these genera were therefore selected for co-culture experiments aimed at stimulating the production of bioactive extracellular compounds. Under co-culture conditions, cell-free secretomes displaying antimicrobial activity were obtained and subsequently subjected to pre-fractionation using acetonitrile (ACN), followed by lyophilization to remove solvents.

**Figure 3:**
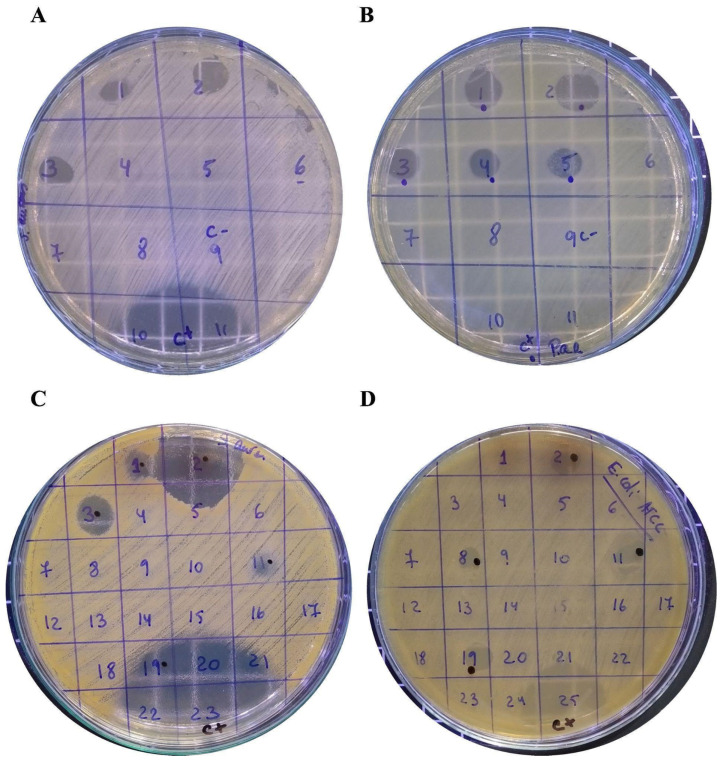
Pre-fractionation of co-culture-derived secretomes and antimicrobial screening of active fractions. Secretomes obtained from bacterial co-cultures were pre-fractionated using SPE-C18 and subsequently lyophilized. Panels (**A**,**B**) show the activity of fractions derived from *S. xiamenensis* LC6 (fractions 1–4; 0–20% ACN/TFA) and *S. putrefaciens* (fractions 5–7; 5–40% ACN/TFA) against a clinical methicillin-resistant *S. aureus* (MRSA) isolate and *P. aeruginosa* ATCC 27853. Panels (**C**,**D**) show the activity of fractions obtained from *Streptomyces* sp. AC-03 against a clinical MRSA isolate and *Escherichia coli* ATCC 11229. Bioactivity was evaluated using spot-on-lawn assays, where clear inhibition zones indicate the presence of extracellular compounds with antimicrobial activity.

The resulting fractions were screened for antimicrobial activity using spot-on-lawn assays. Several fractions inhibited the growth of *Escherichia coli*, *Pseudomonas aeruginosa*, and *Staphylococcus aureus*, as evidenced by the presence of clear inhibition zones (Figure 3A–D). These findings indicated the presence of bioactive compounds within the co-culture-derived secretomes and supported their selection for further purification and characterization.

### 2.6. HPLC Fractionation of Bioactive Secretomes

**Figure 4:**
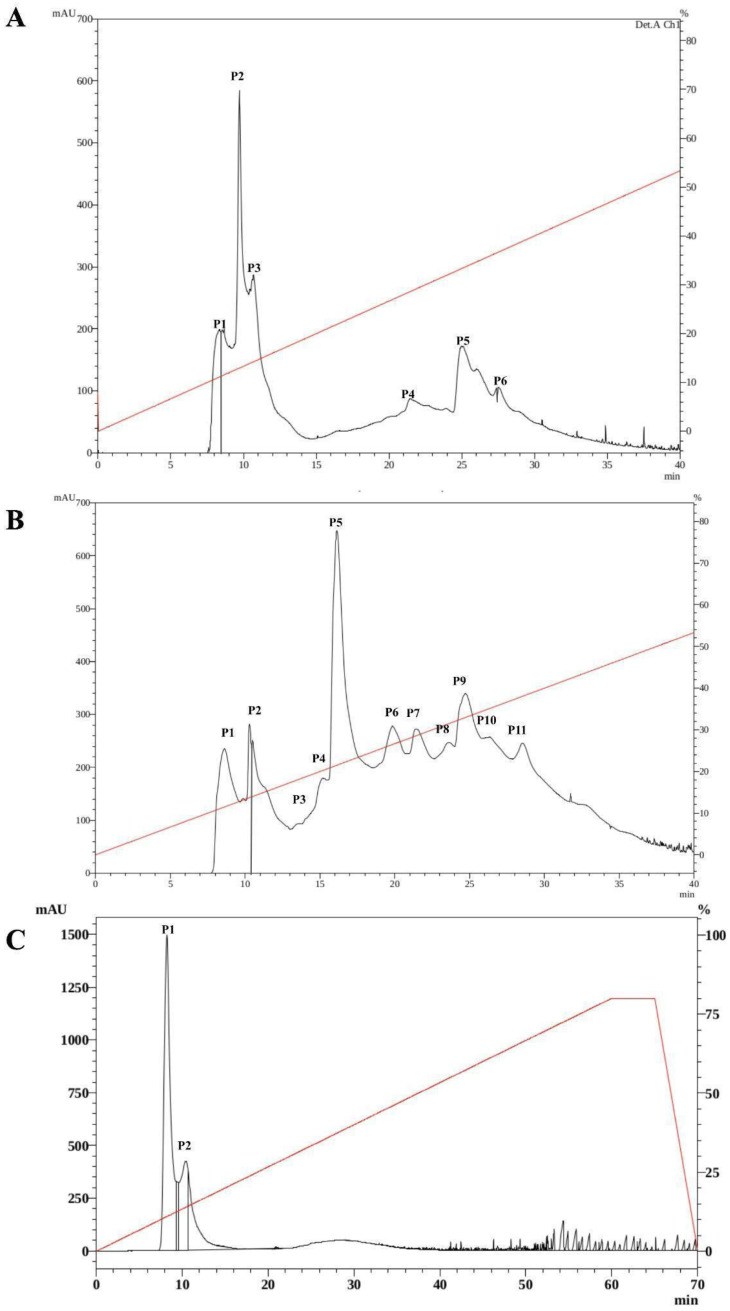
HPLC fractionation of bioactive secretomes. (**A**) Chromatographic profile of the secretome obtained from *S. xiamenensis* LC6. (**B**) Chromatographic profile of *S. putrefaciens*. (**C**) Chromatographic profile of *Streptomyces* sp. AC-03. Separation was performed on a C18 column using an acetonitrile (ACN)/0.05% trifluoroacetic acid (TFA) gradient at a flow rate of 8 mL min^−1^, with detection at 225 nm. Numbered peaks (P1–Pn) indicate the fractions collected for subsequent antimicrobial and peptidomic analyses. Vertical lines indicate the retention time windows used for the collection of fractions P1 and P2. The chromatograms shown are representative of more than three independent biological replicates.

Secretomes displaying antimicrobial activity were further separated by semi-preparative HPLC using a C18 column to obtain fractions associated with the observed bioactivity. The chromatographic profiles obtained for each secretome are shown in Figure 4.

The chromatogram of *S. xiamenensis* LC6 (Figure 4A) revealed six detectable peaks (P1–P6) distributed throughout the ACN/TFA gradient. A prominent peak (P2) was detected during the early stage of the gradient, together with additional peaks exhibiting lower signal intensity (P1 and P3–P6). The observed profile suggests the presence of compounds with different hydrophobic characteristics.

The chromatographic profile of *S. putrefaciens* (Figure 4B) contained eleven resolved peaks (P1–P11) across the gradient. Among these, peak P5 represented the most prominent signal, while several secondary peaks were detected throughout the chromatographic run.

In contrast, the secretome of *Streptomyces* sp. AC-03 (Figure 4C) displayed only two major peaks (P1 and P2), which were subsequently collected and evaluated in downstream analyses.

### 2.7. Antimicrobial and Hemolytic Activities of HPLC-Derived Fractions

**Figure 5:**
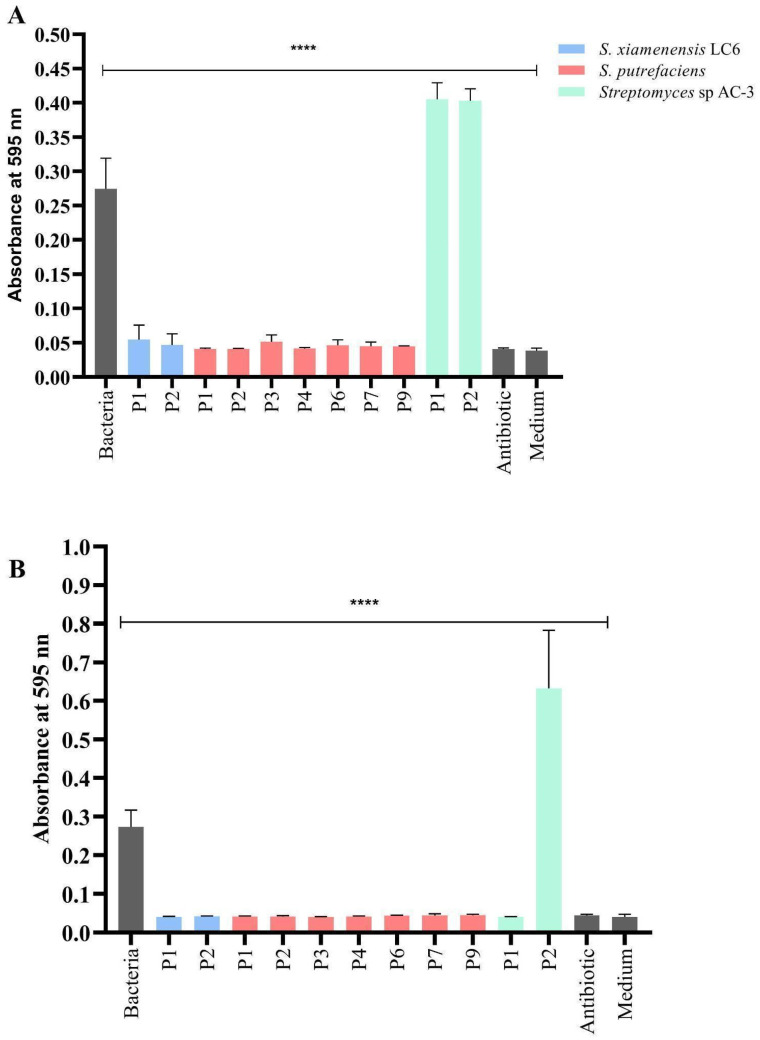
Antimicrobial activity of HPLC-derived fractions. Fractions obtained from *S. xiamenensis* LC6, *S. putrefaciens*, and *Streptomyces* sp. AC-03 were evaluated against *Escherichia coli* SBS 363 (**A**) and *B. megaterium* ATCC 10778 (**B**). Antimicrobial activity was assessed by measuring bacterial growth at 595 nm. Values represent the mean ± standard deviation of three independent experiments (*n* = 3). Statistical significance was determined relative to the untreated control (**** *p* < 0.0001).

The antimicrobial activity of HPLC-derived fractions was evaluated against representative Gram-negative and Gram-positive bacteria. As shown in Figure 5A, the two fractions obtained from *S. xiamenensis* LC6 (P1 and P2) and the seven fractions obtained from *S. putrefaciens* (P1, P2, P3, P4, P6, P7, and P9) inhibited the growth of *Escherichia coli* SBS 363, as evidenced by markedly lower OD_595_ values than the untreated bacterial control. In contrast, neither of the two fractions obtained from *Streptomyces* sp. AC-03 showed inhibitory activity against this microorganism.

When the same fractions were evaluated against *B. megaterium* ATCC 10778 (Figure 5B), all fractions from *S. xiamenensis* LC6 and *S. putrefaciens* remained active. In addition, fraction P1 from *Streptomyces* sp. AC-03 inhibited bacterial growth, whereas fraction P2 did not exhibit detectable activity. Overall, these results indicate that the antimicrobial activity was retained after HPLC fractionation, allowing the selection of bioactive fractions for subsequent characterization.

**Figure 6:**
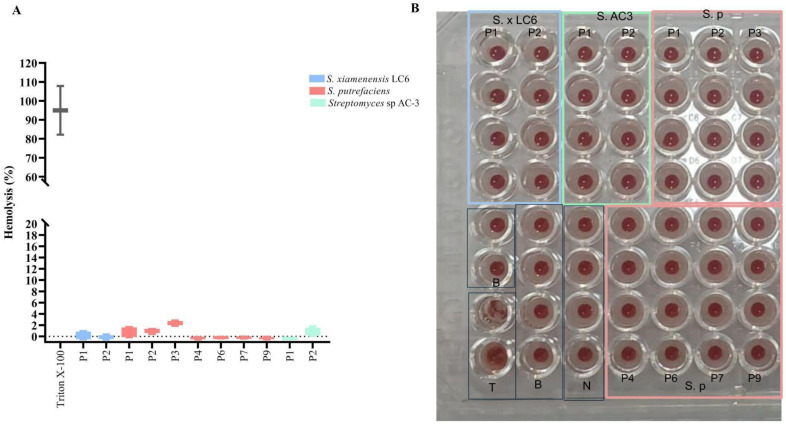
Hemolytic activity of HPLC-derived fractions. (**A**) Percentage of hemolysis induced by fractions obtained from the secretomes of *S. xiamenensis* LC6 (S.x), *S. putrefaciens* (S.p), and *Streptomyces* sp. AC-03 (S AC3). (**B**) Representative erythrocyte microplate assay. Triton X-100 (T) was used as the positive control, PBS (N) as the negative control, and erythrocytes alone served as the blank (B). None of the evaluated fractions exhibited detectable hemolytic activity under the conditions tested.

Hemolytic activity was subsequently evaluated using erythrocytes (Figure 6). None of the analyzed fractions produced detectable hemolysis, and absorbance values were comparable to those of the negative control (PBS). The highest hemolytic activity was observed for Peak 3 derived from *S. putrefaciens*, although the percentage of hemolysis remained below 3%. These results indicate that the bioactive fractions exhibited negligible hemolytic activity under the experimental conditions tested.

### 2.8. Minimum Inhibitory Concentration (MIC)

**Figure 7:**
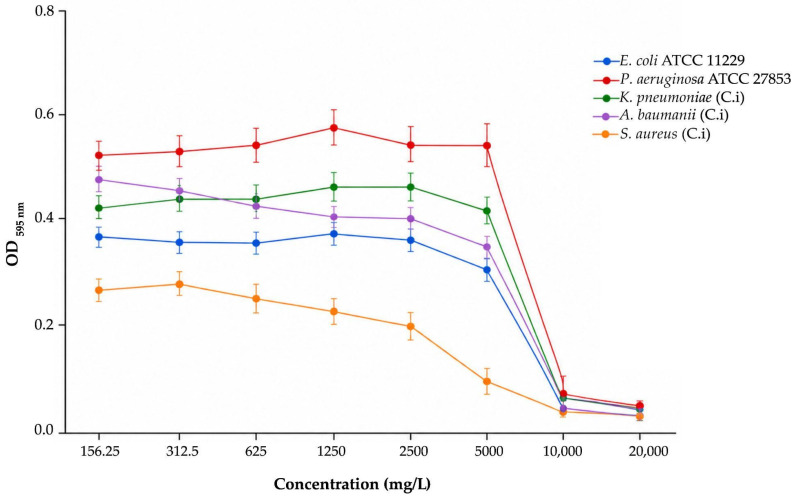
Minimum inhibitory concentration (MIC) assay of the purified bioactive fraction determined by broth microdilution against *Escherichia coli* ATCC 11229, *P. aeruginosa* ATCC 27853, *K. pneumoniae* strain C1, carbapenem-resistant *A. baumannii* (CRAB), and methicillin-resistant *S. aureus* (MRSA). Values represent the mean ± standard deviation of three independent experiments (*n* = 3). Bacterial growth was monitored by measuring OD_595_ following incubation. C.i (Clinical isolation).

The antimicrobial activity of the purified bioactive fraction was evaluated by broth microdilution against reference strains and multidrug-resistant clinical isolates. As shown in Figure 7, bacterial growth inhibition was concentration dependent across all tested microorganisms.

A marked reduction in bacterial growth was observed at concentrations of 10,000 and 20,000 mg/L, where OD_595_ values decreased relative to the untreated controls. Among the tested bacteria, methicillin-resistant *S. aureus* (MRSA) exhibited the highest susceptibility at intermediate concentrations, whereas *P. aeruginosa* ATCC 27853 showed comparatively lower susceptibility. Notably, the purified fraction also inhibited the growth of carbapenem-resistant *A. baumannii* (CRAB), a clinically important multidrug-resistant pathogen.

Overall, the purified bioactive peptide fraction inhibited the growth of both Gram-positive and Gram-negative bacteria, including multidrug-resistant clinical isolates, with an apparent MIC of 10,000 mg/L under the experimental conditions evaluated. This apparent MIC corresponds to the purified bioactive peptide fraction obtained after chromatographic purification and should not be interpreted as the potency of an individual purified peptide.

### 2.9. Antioxidant Activity of Bioactive Fractions

**Figure 8:**
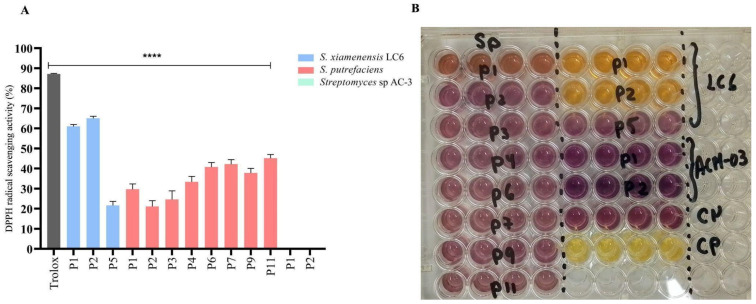
Antioxidant activity of HPLC-derived fractions determined by the DPPH radical scavenging assay. (**A**) Fractions obtained from *S. xiamenensis* LC6, *S. putrefaciens*, and *Streptomyces* sp. AC-03 were evaluated, and Trolox was used as the positive control. Values represent the mean ± SD of three independent experiments (*n* = 3). Statistical analysis was performed using one-way ANOVA followed by Tukey’s multiple-comparison test. Each HPLC-derived fraction was compared with the negative control. Statistical significance is indicated as **** (*p* < 0.0001). (**B**) Representative image of the DPPH radical scavenging assay performed in a 96-well microplate.

The antioxidant activity of HPLC-derived fractions was evaluated using the DPPH radical scavenging assay. As shown in Figure 8, antioxidant activity varied among the fractions obtained from the three bacterial strains. Statistical analysis was performed using one-way ANOVA followed by Tukey’s multiple-comparison test to compare each HPLC-derived fraction with the negative control. Statistical significance was defined as **** (*p* < 0.0001).

Fractions derived from *S. xiamenensis* LC6 exhibited the highest antioxidant activity, with fractions P1 and P2 showing DPPH radical scavenging values of 61.2% and 65.3%, respectively. In contrast, fraction P5 displayed substantially lower activity (22.1%). Fractions obtained from *S. putrefaciens* exhibited moderate antioxidant activity, ranging from 21.4% to 45.6%, with the highest values observed for fractions P6, P7, and P11. No detectable antioxidant activity was observed for fractions derived from *Streptomyces* sp. AC-03.

Overall, the results indicate that several bioactive fractions obtained from *Shewanella* sp. secretomes possess antioxidant properties, whereas the fractions derived from *Streptomyces* sp. AC-03 did not exhibit detectable DPPH radical scavenging activity under the conditions evaluated.

### 2.10. Qualitative Detection of Amino-Containing Compounds

**Figure 9:**
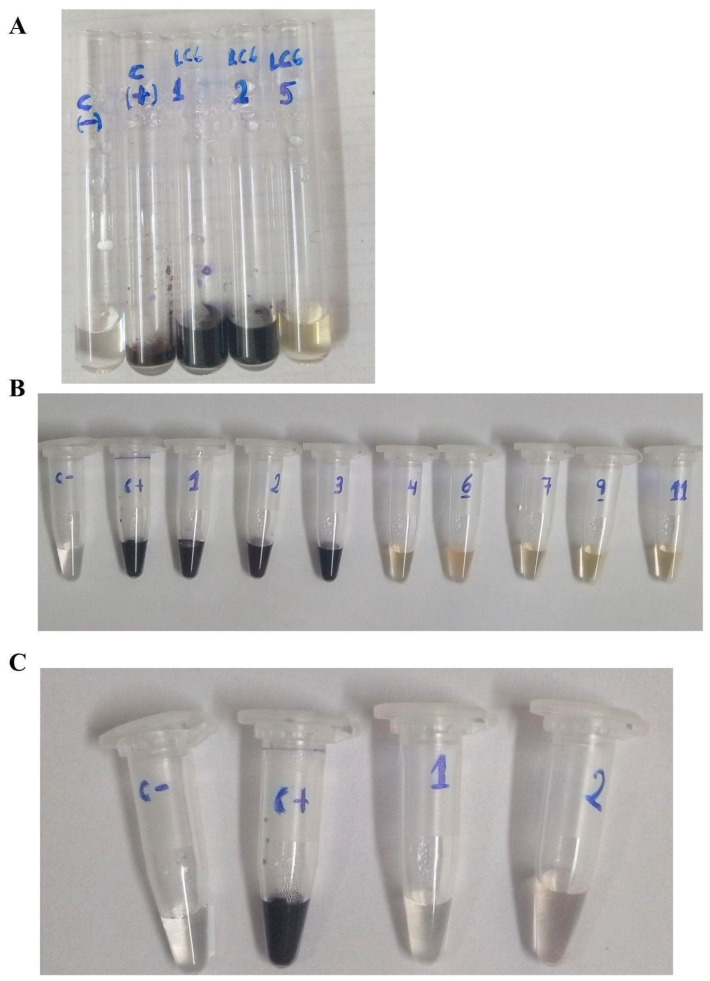
Qualitative screening of amino-containing compounds using the ninhydrin reaction. (**A**) Fractions obtained from the secretome of *S. xiamenensis* LC6, (**B**) fractions obtained from the secretome of *S. putrefaciens*, and (**C**) fractions obtained from the secretome of *Streptomyces* sp. AC-03. Development of a purple coloration indicates the presence of compounds containing free amino groups, whereas weak or absent coloration suggests lower concentrations or the absence of detectable amino-containing compounds under the conditions tested.

Following the antimicrobial activity assays, selected HPLC-derived fractions were subjected to the ninhydrin reaction as a preliminary qualitative screening for compounds containing free amino groups. As shown in Figure 9, several fractions obtained from the secretomes of *S. xiamenensis* LC6 and *S. putrefaciens* developed an intense purple coloration, indicating the presence of amino-containing compounds. In contrast, other fractions exhibited weak coloration or no visible color change, suggesting lower concentrations of such compounds or the absence of accessible free amino groups.

The ninhydrin reaction was included as an initial qualitative assessment and does not provide direct evidence of peptide identity. Nevertheless, the positive reactions observed in selected fractions were consistent with the presence of amino-containing molecules and supported their subsequent characterization by LC-MS/MS analyses.

### 2.11. LC-MS/MS Identification of Peptide Sequences in Active Fractions

**Figure 10:**
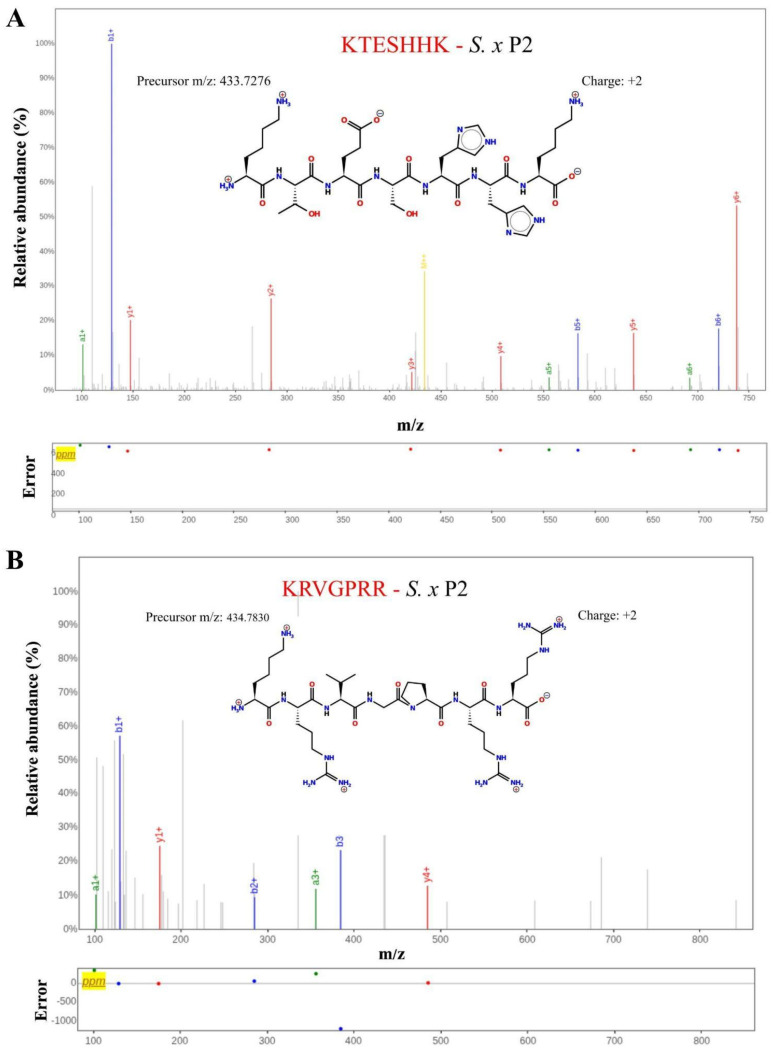
Representative MS/MS spectra and predicted chemical structures of the peptide sequences KTESHHK (**A**) and KRVGPRR (**B**) detected in the active fraction S.x-P2 obtained from *S. xiamenensis* LC6. Peptide sequences were assigned by LC-MS/MS and de novo sequencing.

**Figure 11:**
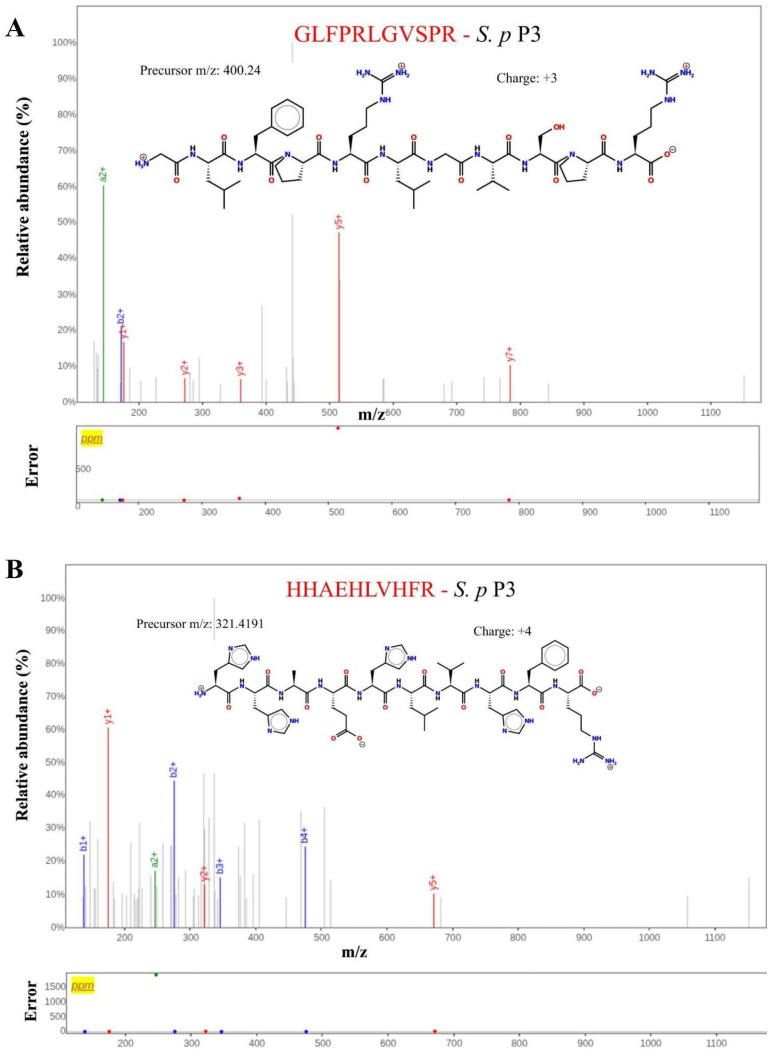
Representative MS/MS spectra and predicted chemical structures of the peptide sequences GLFPRLGVSPR (**A**) and HHAEHLVHFR (**B**) detected in the active fraction S.p-P3 obtained from *S. putrefaciens*. Peptide sequences were assigned by LC-MS/MS and de novo sequencing.

LC-MS/MS analysis of active fractions obtained from *S. xiamenensis* LC6 (S.x-P2) and *S. putrefaciens* (S.p-P3) enabled the detection of several peptide sequences through de novo sequencing. Representative peptide sequences identified in fraction S.x-P2 included KTESHHK and KRVGPRR (Figure 10), whereas GLFPRLGVSPR and HHAEHLVHFR were selected as representative sequences from fraction S.p-P3 (Figure 11).

KTESHHK and KRVGPRR consisted of seven amino acid residues and exhibited precursor ions with *m*/*z* values of 433.7276 and 434.7830, respectively. GLFPRLGVSPR and HHAEHLVHFR contained 11 and 10 amino acid residues and displayed precursor ions with *m*/*z* values of 400.24 and 321.4191, respectively. The corresponding MS/MS spectra showed characteristic b- and y-ion fragmentation patterns that supported sequence assignment through de novo analysis.

Peptide sequences were detected within chromatographic fractions that retained antimicrobial activity after purification, providing evidence for the presence of peptide-containing molecules in the analyzed secretomes.

### 2.12. Predicted Biosynthetic Gene Clusters

**Figure 12:**
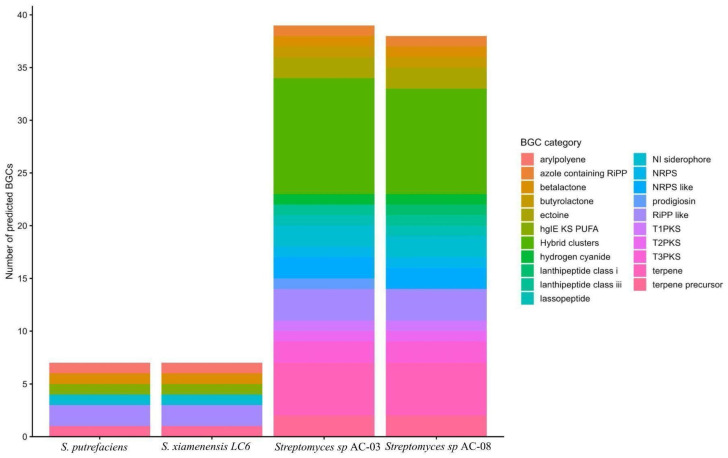
Distribution of predicted biosynthetic gene clusters (BGCs) identified in *S. xiamenensis* LC6, *S. putrefaciens*, and *Streptomyces* sp. strains AC-03 and AC-08. BGCs were predicted using antiSMASH and classified according to their biosynthetic category.

Genome mining identified seven predicted biosynthetic gene clusters (BGCs) in both *S. xiamenensis* LC6 and *S. putrefaciens*. The BGC repertoires of the two strains were similar and included clusters classified as arylpolyenes, siderophores, RiPP-like compounds, terpene precursors, betalactones, and hglE-KS/PUFA-associated clusters (Figure 12).

In contrast, *Streptomyces* sp. strains AC-03 and AC-08 contained 39 and 38 predicted BGCs, respectively (Supplementary Table S5). These genomes harbored a greater diversity of biosynthetic pathways, including non-ribosomal peptide synthetases (NRPS), NRPS-like clusters, siderophores, RiPP-like compounds, terpenes, lanthipeptides, type III polyketide synthases (T3PKS), betalactones, lassopeptides, and several hybrid BGCs (Figure 12).

Overall, the genomic analyses revealed a broader biosynthetic potential in the Streptomyces strains compared with the *Shewanella* isolates, consistent with the recognized capacity of actinomycetes to produce structurally diverse secondary metabolites.

## 3. Discussion

Bioprospecting of molecules with antimicrobial activity has become a global priority in response to the growing antimicrobial resistance (AMR) crisis, which demands the development of new therapeutic agents. These include natural products, drug repurposing strategies, antimicrobial peptides (AMPs) from diverse biological sources, and alternative approaches such as bacteriophage therapy to combat infections that are increasingly difficult to treat [4,12].

Extreme environments characterized by unusual conditions of temperature, salinity, pH, or radiation represent reservoirs of microorganisms with unique metabolic adaptations and considerable yet largely unexplored biotechnological potential, including among non-cultivable species [21]. In South America, the diversity of these environments suggests the existence of microbial communities with distinctive metabolic capabilities shaped by ecological competition and environmental selective pressures [22,23]. Consequently, bioprospecting in megadiverse regions such as South America represents an attractive strategy for identifying novel bioactive molecules with antimicrobial potential.

Our analysis of 853 South American bacterial genomes revealed a heterogeneous distribution of taxa associated with predicted AMP candidates, including genera frequently linked to the production of secondary metabolites, such as *Streptomyces*, *Bacillus*, *Burkholderia*, and *Shewanella* (Supplementary Figure S1). These findings are consistent with previous reports describing the ability of these bacterial groups to produce antimicrobial compounds and ribosomally synthesized and post-translationally modified peptides (RiPPs) [24,25,26,27]. The application of an AMP probability threshold ≥ 0.6 enabled the prioritization of peptide candidates for subsequent analyses, a strategy comparable to recent genome-mining studies that employ machine learning approaches to facilitate the identification of promising AMP candidates from large sequencing datasets [18].

Metagenomic analyses revealed a broad diversity of environmental sources (Supplementary Figure S2), including marine sediments, wastewater, soils, and freshwater systems. These ecosystems represent complex microbial communities that may constitute extensive reservoirs of biosynthetic diversity. Previous studies have shown that environments exposed to strong selective pressures, including hydrocarbon contamination and other environmental stresses, frequently exhibit enrichment of genes associated with secondary metabolism and antimicrobial compound production [10]. Therefore, the exploration of environmental genomes and metagenomes from South America provides access to a largely untapped source of microbial diversity with potential relevance for antimicrobial discovery.

Recent developments in artificial intelligence and machine learning have also facilitated the prediction of additional biological properties, including toxicity, hemolytic behavior, and potential therapeutic functions [15]. As summarized in Supplementary Tables S3 and S4, the computational workflow employed in this study enabled the prioritization of peptide candidates with predicted antimicrobial activity, low toxicity, and non-hemolytic profiles. Several candidates were additionally predicted to possess anti-inflammatory and anticancer properties, although these activities remain computational predictions and require experimental validation.

Interestingly, several bioactive fractions exhibited antioxidant activity in the DPPH assay. Because oxidative stress and inflammatory responses are closely interconnected through the generation of reactive oxygen species (ROS), the coexistence of antimicrobial activity, antioxidant activity, and predicted anti-inflammatory properties merits further investigation. Nevertheless, these observations should be interpreted with caution until the responsible molecules are identified and their biological activities are experimentally confirmed.

Structural modeling of the prioritized peptide candidates revealed a predominance of α-helical conformations (Figure 1 and Figure 2). This structural feature is frequently associated with antimicrobial peptides because it facilitates interactions with bacterial membranes through electrostatic attraction and hydrophobic insertion into the lipid bilayer [27,28]. Although the structural models generated by AlphaFold3 remain computational predictions, the prevalence of α-helical motifs among the selected candidates is consistent with structural characteristics commonly reported for antimicrobial peptides.

Microbial interactions generate chemical signals and competitive responses that can stimulate the production of secondary metabolites not expressed under monoculture conditions [29,30]. In this context, bacterial co-culture has emerged as a valuable strategy for activating silent biosynthetic pathways and expanding the diversity of detectable bioactive metabolites. In the present study, secretomes obtained from co-cultures involving *S. xiamenensis* LC6, *S. putrefaciens*, and *Streptomyces* sp. AC-03 exhibited antimicrobial activity against both Gram-positive and Gram-negative model bacteria. These observations support the role of interspecies interactions in stimulating the production of bioactive extracellular compounds.

Similar findings have been reported in actinomycetes, where co-culture approaches enhanced antimicrobial activity against methicillin-resistant *S. aureus* (MRSA) [31]. Consistent with these reports, fractions obtained by solid-phase extraction (SPE) from the co-culture-derived secretomes displayed activity against a clinical MRSA isolate. Subsequent HPLC fractionation yielded multiple bioactive fractions that retained antimicrobial activity, further supporting the effectiveness of co-culture-based approaches for identifying biologically active metabolites.

The *Shewanella* secretomes analyzed in this study exhibited activity against Gram-negative bacteria (Figure 3A,B), including *P. aeruginosa* ATCC 27853, a strain recognized for its biofilm-forming capacity. Following HPLC fractionation, several peaks retained antimicrobial activity against *Escherichia coli*, indicating that the active components remained detectable after the purification process. Previous studies have also reported the production of antimicrobial metabolites by members of the genus *Shewanella* [26]. In addition, extracellular metabolites produced by *S. putrefaciens* have been associated with postbiotic properties, including biofilm inhibition and antiviral activity, while exhibiting low cytotoxicity [32].

These observations are consistent with the results obtained from the *S. putrefaciens* strain isolated from textile effluent environments. In our study, secretome-derived fractions inhibited the growth of *Escherichia coli* and *P. aeruginosa*, while HPLC fractionation generated additional bioactive fractions that retained activity against *Escherichia coli* SBS 363 and *Bacillus megaterium*. Together, these findings support the potential of *Shewanella* secretomes as a source of biologically active extracellular molecules deserving further characterization.

The absence of detectable hemolytic activity in the evaluated fractions (Figure 6) suggests a favorable selectivity profile toward bacterial cells, an important characteristic for the development of antimicrobial compounds. These findings, together with the low toxicity predicted for several prioritized peptide candidates, support their further evaluation in future biological studies. Nevertheless, the biological activities of individual peptide candidates remain to be experimentally validated.

In addition to their predicted antimicrobial properties, several peptide candidates identified through genome and metagenome mining were also predicted to possess anti-inflammatory and anticancer activities. These functions were inferred exclusively from computational analyses and should therefore be considered preliminary predictions. Similar multifunctional properties have been described for several antimicrobial peptides, including the bacteriocin Pediocin PA-1, which has been reported to modulate immune responses and exert anticancer effects [33]. Consequently, the identification of peptide candidates with multiple predicted biological activities provides additional information for candidate prioritization. These computational predictions require experimental validation and should be regarded as a basis for future studies rather than confirmed biological activities.

The antimicrobial activity observed during the initial screening was further supported by broth microdilution assays performed with the purified bioactive fraction (Figure 7). An apparent MIC of 10,000 mg/L was observed against all tested microorganisms, including multidrug-resistant clinical isolates and carbapenem-resistant *A. baumannii*. The retention of antimicrobial activity following chromatographic purification indicates that biologically active molecules remained present within the purified bioactive fractions and supports their contribution to the antimicrobial activity detected in the secretomes. These findings provide a foundation for future studies aimed at the purification of individual peptide components, peptide synthesis, and functional validation to determine their specific contribution to the observed antimicrobial activity and to evaluate their translational potential.

Several fractions also exhibited DPPH radical scavenging activity (Figure 8), with the highest values observed for fractions P1 and P2 derived from *S. xiamenensis* LC6. Reactive oxygen species (ROS) play a central role in oxidative stress and are closely associated with inflammatory processes. Therefore, the antioxidant activity detected in these fractions may represent an additional biological property of the secretome-derived molecules. Previous studies have reported that bioactive peptides capable of scavenging free radicals may also contribute to the modulation of oxidative stress-associated inflammatory responses [34,35]. Although anti-inflammatory activity was not experimentally evaluated in the present study, the observed antioxidant activity suggests that these fractions may contain molecules with additional biological properties that warrant further investigation. However, because these activities were evaluated using bioactive chromatographic fractions rather than isolated compounds, the specific molecules responsible for the observed antioxidant activity remain to be identified. Further purification and functional characterization of individual peptide components will be required to establish their specific biological contributions.

The ninhydrin assay was included as a preliminary qualitative screening to assess the presence of amino-containing compounds in the active fractions and was not intended to provide evidence of peptide identity, which was subsequently investigated by LC-MS/MS. The purple coloration observed in several fractions derived from *S. xiamenensis* LC6 and *S. putrefaciens* (Figure 9) indicates the presence of compounds containing free amino groups, consistent with amino acid or peptide containing molecules. Previous studies have employed the ninhydrin reaction in combination with complementary analytical approaches to characterize extracellular antibacterial substances, noting that weak or absent reactions may occur in cyclic peptides or molecules with limited accessibility of free amino groups [36]. Therefore, although the ninhydrin assay does not identify peptides directly, the positive reactions observed in selected fractions were consistent with the presence of amino-containing compounds and supported their subsequent characterization by LC-MS/MS.

LC-MS/MS analyses of the active fractions enabled the detection of several peptide sequences through de novo sequencing. Representative sequences identified in fractions S.x-P2 and S.p-P3 consisted of short peptides ranging from 7 to 11 amino acid residues. The detection of these sequences within chromatographic fractions that retained antimicrobial activity after purification provides evidence that peptide-containing molecules were present in the analyzed secretomes of *Shewanella* spp. Although the biological activities of individual peptides remain to be experimentally determined, their identification represents an important step toward characterizing the molecular composition of the bioactive fractions.

The peptide sequences identified by LC-MS/MS did not completely overlap with the AMP candidates predicted by the genome and metagenome mining analyses. This difference is expected because genome and metagenome mining predict the biosynthetic potential encoded in microbial genomes, whereas LC-MS/MS detects peptides that are produced under the specific co-culture conditions evaluated [37,38]. In addition, peptide maturation, post-translational processing, degradation during secretion or sample preparation, and the current limitations of AMP prediction algorithms may also contribute to these differences [39]. Therefore, genome mining and peptidomic analyses should be regarded as complementary approaches for antimicrobial peptide discovery rather than methods expected to produce identical results.

Although a direct correspondence between the computationally predicted AMP candidates and the peptide sequences detected by LC-MS/MS could not be established. Nevertheless, the presence of peptide-containing molecules within fractions that exhibited antimicrobial activity provides preliminary experimental support for the computational screening strategy and highlights candidate molecules for future functional characterization. Additional studies involving peptide synthesis and biological validation will be required to determine the contribution of individual peptides to the observed activities.

The genome mining analysis of *S. xiamenensis* LC6 and *S. putrefaciens* revealed a conserved repertoire of biosynthetic gene clusters (BGCs) (Figure 12), suggesting a relatively similar biosynthetic potential within these species. Although members of the genus *Shewanella* have been extensively investigated for applications in bioremediation and metal reduction [40,41], their capacity to produce antimicrobial secondary metabolites remains comparatively underexplored. Among the predicted BGCs, RiPP-like and betalactone-associated clusters appear particularly interesting because related pathways have been associated with the production of antimicrobial compounds and enzyme inhibitors in other bacterial taxa [42,43]. In contrast, terpene, arylpolyene, and PUFA-associated clusters are more commonly linked to physiological functions such as protection against oxidative stress rather than direct antimicrobial activity (Supplementary Tables S5 and S6) [44,45].

The *Streptomyces* strains exhibited a substantially larger biosynthetic repertoire, harboring nearly 40 predicted BGCs (Figure 12). This observation is consistent with the recognized role of *Streptomyces* species as prolific producers of structurally diverse secondary metabolites [46]. Notably, many of these clusters corresponded to hybrid biosynthetic systems, which are frequently associated with complex metabolic pathways and enhanced chemical diversity [47]. The abundance and diversity of predicted BGCs further support the biosynthetic potential of these strains as sources of bioactive metabolites.

## 4. Materials and Methods

### 4.1. Data Retrieval

Bacterial genomic and metagenomic datasets were retrieved from the National Center for Biotechnology Information (NCBI) databases, including assembled genomes, contigs, and Sequence Read Archive (SRA) datasets. Searches were conducted using combinations of the keywords “Peru”, “Ecuador”, “Chile”, “Argentina”, “Colombia”, “Brazil”, “environmental”, “soil”, “marine”, and “river”. Data retrieval and downloads were performed between October and December 2024.

Assembled genomes were downloaded in FASTA format (.fasta or .fna). For datasets available only as raw sequencing data, corresponding FASTQ files were obtained from the SRA database for subsequent assembly and analysis. Metadata associated with all genomic and metagenomic datasets analyzed in this study are provided in Supplementary Table S1.

### 4.2. Genome and Metagenome Assembly

Genomic and metagenomic datasets retrieved as raw sequencing reads from the Sequence Read Archive (SRA) were assembled using SPAdes [48] through the BV-BRC platform (https://www.bv-brc.org/, accessed on 22 October 2024). The assembly workflow implemented by the platform included read normalization, trimming of low-quality short reads, filtering of long reads, de novo assembly, and polishing steps.

For assembly optimization, the workflow used an estimated genome size of 5 Mb and a target coverage of 200× when applicable. Assembly polishing consisted of two iterations of Racon followed by two iterations of Pilon. To improve assembly quality, contigs shorter than 300 bp or with coverage values below 5× were excluded from downstream analyses. The resulting contig.fasta files were subsequently used for antimicrobial peptide candidate prediction and further bioinformatic analyses.

### 4.3. Identification of Antimicrobial Peptide Candidates in Genomes and Metagenomes

Antimicrobial peptide (AMP) candidate prediction was performed using the machine learning-based models implemented in Macrel [49], a tool specifically developed for the identification of putative AMP candidates from genomic and metagenomic sequences.

To facilitate large-scale analyses, a custom Bash script was developed to automate the execution of Macrel across multiple datasets (available at: https://github.com/Abraham-Culupu, accessed on 10 February 2026). For each genome or metagenome analyzed, predicted peptide sequences and their associated AMP probability scores were consolidated into a single output file. Peptide candidates with AMP probability scores ≥ 0.6 were retained for subsequent analyses.

Retained candidates were further evaluated using complementary computational tools to predict antimicrobial activity, hemolytic behavior, toxicity, anti-inflammatory activity, anticancer activity, physicochemical properties, and structural features. Peptide candidates selected for structural modeling corresponded to those exhibiting the most favorable combination of predicted antimicrobial activity, low toxicity, non-hemolytic behavior, and physicochemical characteristics.

### 4.4. Prediction of Antimicrobial Activity

The antimicrobial potential of peptide candidates was evaluated using several machine learning-based prediction tools, including iAMPpred [50], AMP Scanner v2 [51], and CAMPR3 [52]. These platforms employ different classification algorithms, including Support Vector Machines (SVM), Random Forest (RF), Artificial Neural Networks (ANN), and Discriminant Analysis, to estimate the probability of peptides exhibiting antimicrobial activity.

For AMP Scanner v2, peptide sequences were submitted in FASTA format using the v2 Feb2020 prediction model, which evaluates peptides ranging from 10 to 200 amino acids in length. Peptides with prediction probabilities > 0.5 were classified as AMP candidates. For CAMPR3, all available prediction algorithms (SVM, RF, ANN, and Discriminant Analysis) were applied, and peptides with prediction probabilities > 0.5 were also considered AMP candidates. Predictions generated by these tools were used as complementary criteria for peptide candidate prioritization.

### 4.5. Prediction of Anti-Inflammatory Activity and Toxicity

The potential anti-inflammatory activity of peptide candidates was evaluated using PreAIP [53] and PepNet [54]. Peptide toxicity was assessed using ToxinPred3 [55], a machine learning-based platform that classifies peptides as toxic or non-toxic based on sequence-derived features.

For PreAIP, peptide sequences were submitted in FASTA format and analyzed using the single prediction mode. For PepNet, the anti-inflammatory peptide (AIP) prediction model was selected, and analyses were performed using the fast mode. Toxicity predictions were conducted using ToxinPred3 with the ET-based prediction model. Peptides with prediction scores above 0.5 were classified according to their predicted toxicity profiles. The results obtained from these analyses were used as complementary criteria for peptide candidate prioritization.

### 4.6. Prediction of Non-Hemolytic Peptides

The hemolytic potential of peptide candidates was evaluated using HemoPI-2 [56]. Peptide classification was performed using the Hybrid1 (HIB1) prediction model, which integrates multiple sequence-based features to discriminate hemolytic from non-hemolytic peptides. A threshold value of 0.5 was applied according to the platform recommendations. Peptides predicted as non-hemolytic were prioritized for subsequent analyses.

### 4.7. Prediction of Anticancer Activity

The potential anticancer activity of peptide candidates was evaluated using AntiCP 2.0 [57]. To complement these analyses, mACPpred 2.0 [58] was also employed for anticancer peptide prediction. Peptide sequences were analyzed using the recommended prediction settings for each platform, and the results were incorporated as complementary criteria for peptide candidate prioritization.

### 4.8. Physicochemical Property Analysis and Structural Prediction

The amino acid composition, molecular weight, and theoretical isoelectric point (pI) of peptide candidates were calculated using PepCalc (https://www.pep-calc.com/, accessed on 20 February 2025) [59]. Additional physicochemical parameters, including the instability index, aliphatic index, and grand average of hydropathicity (GRAVY), were calculated using ProtParam (https://web.expasy.org/protparam/, accessed on 20 February 2025).

Three-dimensional structural models of selected peptide candidates were generated from their primary amino acid sequences using the AlphaFold3 server (https://alphafoldserver.com/, accessed on 20 March 2025). Peptides selected for structural modeling corresponded to candidates exhibiting favorable predicted antimicrobial activity, low toxicity, non-hemolytic behavior, and suitable physicochemical characteristics.

### 4.9. Prediction of Biosynthetic Gene Clusters

Biosynthetic gene clusters (BGCs) associated with secondary metabolite production were predicted in the genomes of *S. xiamenensis* LC6, *S. putrefaciens*, and two *Streptomyces* strains using antiSMASH v8.0 [60]. Analyses were performed using the standard antiSMASH workflow with relaxed detection strictness enabled.

BGCs predicted to contain more than one core biosynthetic category were classified as hybrid clusters for comparative analyses. Predicted clusters were subsequently grouped according to their biosynthetic class and compared among the analyzed bacterial strains.

### 4.10. Selection and Reactivation of Bacterial Strains

Based on the genome-mining analyses and the availability of environmental isolates in the strain collection of the Laboratory of Molecular Microbiology and Biotechnology, selected bacterial strains were reactivated for experimental evaluation. The analyzed microorganisms included *S. xiamenensis* LC6, *S. putrefaciens* (isolated from textile industry effluents), and *Streptomyces* spp.

*Shewanella* strains were cultured in tryptic soy broth (TSB), whereas *Streptomyces* strains were cultivated in Czapek broth. Cultures were maintained under standard laboratory growth conditions prior to subsequent co-culture and bioactivity assays.

### 4.11. Bacterial Co-Culture

To stimulate the production of bioactive metabolites, a bacterial co-culture approach was employed. This strategy has been widely used to activate secondary metabolic pathways and promote the production of compounds that may not be expressed under monoculture conditions [19].

Environmental isolates of *S. xiamenensis* LC6, *S. putrefaciens*, and *Streptomyces* spp. were cultured individually until reaching an optical density of OD600 = 0.4. Subsequently, each culture was co-incubated with *Escherichia coli* ATCC 11229 and a clinical methicillin-resistant *S. aureus* (MRSA) isolate by adding 200 µL of indicator cultures at the death phase, resulting in an approximate producer-to-indicator ratio of 250:1 (*v*/*v*). Indicator cultures were intentionally added at the death phase to promote competitive interactions and induce the production of bioactive metabolites by the producer strains.

Co-cultures were established in 50 mL of the corresponding culture medium and incubated at 30 °C with shaking at 120 rpm for 48–60 h, depending on the bacterial strain. *Shewanella* co-cultures were typically incubated for 48 h, whereas Streptomyces co-cultures were maintained for up to 60 h to allow adequate metabolite production. The incubation conditions were adapted from those described by Sung [20].

### 4.12. Pre-Fractionation of Co-Culture Supernatants

Co-cultures were centrifuged at 7800 rpm for 20 min at 4 °C to remove bacterial cells. The resulting supernatants were filtered through 0.2 µm membrane filters using a vacuum filtration system to obtain cell-free extracts. Filtered supernatants were acidified with glacial acetic acid to a final concentration of 1 M and incubated at 4 °C for 1 h under agitation, following previously described procedures [61,62].

Samples were subsequently ultracentrifuged at 30,000 rpm for 1 h at 4 °C to remove residual particulate material and obtain clarified supernatants. Clarified supernatants were subjected to solid-phase extraction (SPE) using Sep-Pak C18 cartridges (5 g; Waters Corp., Milford, MA, USA). Elution was performed with increasing concentrations of acetonitrile containing 0.05% trifluoroacetic acid (TFA) (5%, 10%, 20%, 40%, and 80%). The collected fractions were lyophilized and resuspended in ultrapure water prior to subsequent analyses.

### 4.13. Antimicrobial Activity Assays

The antimicrobial activity of the obtained fractions was evaluated against *Escherichia coli* ATCC 11229, *P. aeruginosa* ATCC 27853, and a clinical methicillin-resistant Staphylococcus aureus (MRSA) isolate using the spot-on-lawn assay. Briefly, bacterial suspensions adjusted to approximately 0.5 McFarland standard were uniformly spread onto the surface of agar plates. Subsequently, 20 µL of each fraction was deposited onto the inoculated agar surface and allowed to absorb.

Plates were incubated at 37 °C for 18–24 h, and antimicrobial activity was determined by the presence of visible inhibition zones surrounding the applied fractions. All assays were performed in triplicate.

### 4.14. HPLC Fractionation and Evaluation of Antimicrobial and Hemolytic Activities

Supernatants and pre-fractions exhibiting antimicrobial activity were further fractionated using a semi-preparative HPLC system (Shimadzu LC-8A system, Shimadzu Corporation, Kyoto, Japan) equipped with a C18 column (300 Å, 250 × 10 mm). Elution was performed using acetonitrile containing 0.05% trifluoroacetic acid (TFA) as the mobile phase at a flow rate of 8 mL/min, and chromatograms were monitored at 225 nm. Eluted peaks were manually collected, frozen, and lyophilized for subsequent biological assays and peptidomic analyses.

Lyophilized HPLC fractions were reconstituted in ultrapure water to a final concentration of 100 g/L. Antimicrobial activity was evaluated in 96-well microplates following the procedure described by Espinoza-Culupú [63]. Briefly, 20 µL of each fraction was tested against the selected bacterial strains, and antimicrobial activity was assessed by measuring inhibition of bacterial growth. All assays were performed in triplicate.

Hemolytic activity was evaluated as described by Thiago [64]. Erythrocytes were washed with phosphate-buffered saline (PBS) and resuspended at a final concentration of 3% (*v*/*v*). Aliquots of 50 µL of erythrocyte suspension were incubated with individual HPLC fractions at 37 °C for 3 h. Hemolysis was quantified by measuring absorbance at 405 nm. Triton X-100 (0.1%) and PBS were used as positive and negative controls, respectively. All experiments were performed in triplicate.

### 4.15. Minimum Inhibitory Concentration (MIC) Assay

The antimicrobial activity of the purified bioactive fraction was evaluated using a broth microdilution assay following previously described methodologies for antimicrobial peptide evaluation [65], with minor modifications. Lyophilized fractions were resuspended in ultrapure water at an initial concentration of 100 g/L and subjected to two-fold serial dilutions (20,000–156 mg/L) in Mueller-Hinton broth using sterile 96-well microplates with a final volume of 100 µL per well.

The assays were performed against *Escherichia coli* ATCC 11229, *P. aeruginosa* ATCC 27853, *K. pneumoniae* C1 (multidrug-resistant clinical isolate), carbapenem-resistant *A. baumannii* (CRAB), and methicillin-resistant *S. aureus* (MRSA). Plates were incubated at 37 °C for 18–24 h, and bacterial growth was monitored by measuring optical density at 595 nm using a microplate reader.

The apparent minimum inhibitory concentration (MIC) was defined as the lowest concentration associated with marked growth inhibition relative to the untreated growth control. All experiments were performed in triplicate.

### 4.16. Determination of Antioxidant Activity by the DPPH Radical Scavenging Assay

The antioxidant activity of HPLC-derived fractions was evaluated using the 2,2-diphenyl-1-picrylhydrazyl (DPPH) radical scavenging assay as previously described by Espinoza-Culupú [35], with minor modifications. Briefly, 20 µL of each fraction (100 g/L) was mixed with 180 µL of a freshly prepared DPPH solution (0.2 mM) in a sterile 96-well microplate.

Trolox was used as the positive control, whereas the reaction mixture without sample served as the negative control. The mixtures were incubated in the dark at 37 °C for 30 min, and absorbance was measured at 517 nm using a microplate reader. Antioxidant activity was expressed as the percentage of DPPH radical scavenging relative to the negative control. All assays were performed in triplicate.

### 4.17. Qualitative Screening of Amino-Containing Compounds by the Ninhydrin Reaction

HPLC-derived fractions resuspended in ultrapure water were qualitatively evaluated for the presence of compounds containing free amino groups using the ninhydrin reaction [66]. A ninhydrin solution (3.5 mg/mL) was prepared in ethanol and mixed with each fraction at a 1:1 (*v*/*v*) ratio (50 µL of fraction and 50 µL of ninhydrin solution).

Samples were incubated at 95 °C for 10 min, and color development was visually assessed. The appearance of a purple coloration was considered indicative of the presence of compounds containing free amino groups, whereas weak or absent coloration suggested lower concentrations or the absence of detectable amino-containing compounds under the conditions tested.

### 4.18. Peptide Sequencing by LC-MS/MS

Fractions exhibiting antimicrobial activity after HPLC fractionation were selected for peptidomic analyses. Specifically, fraction P2 from *S. xiamenensis* LC6 (S.x-P2), fractions P3 and P4 from *S. putrefaciens* (S.p-P3 and S.p-P4), and fractions P1 and P2 from *Streptomyces* sp. AC-03 (AC-03-P1 and AC-03-P2) were analyzed by LC-MS/MS. These bioactive fractions were subjected to additional sample preparation, including peptide quantification using a bovine serum albumin (BSA) peptide standard, filtration through 10 kDa Amicon^®^ Ultra centrifugal filter units (Merck Millipore, Burlington, MA, USA) to enrich low-molecular-weight peptides (<10 kDa) and remove higher-molecular-weight proteins, followed by StageTip purification prior to LC-MS/MS analysis.

Peptide fractions were purified using SDB-XC resin-packed StageTip microcolumns according to the protocol described by Rappsilber [67]. Retained peptides were washed with 4% acetonitrile containing 0.1% trifluoroacetic acid (TFA) and subsequently eluted with 80% acetonitrile containing 0.1% TFA. Eluates were concentrated using a SpeedVac system and resuspended prior to analysis.

Peptide sequencing was performed at the Laboratório de Proteômica e Espectrometria de Massas of the Laboratório Especial de Toxinologia Aplicada (LETA), Butantan Institute, São Paulo, Brazil. Analyses were conducted using an Orbitrap Exploris 480 mass spectrometer (Thermo Fisher Scientific, Bremen, Germany) coupled to a Vanquish Neo nanoLC system (Thermo Fisher Scientific), following peptidomic workflows similar to those described by Rasslan [68]. Raw LC-MS/MS data were subsequently processed using de novo peptide sequencing approaches and complementary bioinformatic analyses for peptide sequence identification.

### 4.19. Bioinformatic Analysis of Peptide Sequences

Raw LC-MS/MS data were analyzed using the Novor Cloud platform (Rapid Novor Inc., Kitchener, ON, Canada (https://app.novor.cloud/, accessed on 30 April 2026)) for de novo peptide sequencing. The resulting peptide sequences were used for downstream bioinformatic analyses and comparison with available genomic and transcriptomic resources.

Peptide sequences identified in fraction S.x-P2 were compared against the genomic and transcriptomic datasets of *S. xiamenensis* LC6 available under BioProject PRJNA547647. Similarly, peptide sequences identified in fractions S.p-P3 and S.p-P4 were compared against the genome sequence of *S. putrefaciens* generated in an independent study. Peptides identified in fractions AC-03-P1 and AC-03-P2 were compared against the genome sequence of *Streptomyces* sp. AC-03.

Sequence comparisons were performed to identify genomic regions and predicted open reading frames (ORFs) potentially associated with the peptide sequences detected by LC-MS/MS. Two-dimensional representations of selected peptide sequences were generated using the PepDraw web platform (https://pepdraw.com/app, accessed on 1 May 2026) for structural visualization.

### 4.20. Whole-Genome Sequencing

Genomic DNA from *Shewanella* and *Streptomyces* strains was extracted from logarithmic-phase cultures using the GenElute Bacterial Genomic DNA Kit (Sigma-Aldrich, St. Louis, MO, USA) according to the manufacturer’s instructions. DNA concentration and purity were evaluated using a NanoDrop spectrophotometer based on the absorbance ratios at 260/280 nm and 260/230 nm. DNA integrity was verified by agarose gel electrophoresis.

Whole-genome sequencing was performed using an Illumina MiSeq platform (Faculty of Biological Sciences, Universidad Nacional Mayor de San Marcos, Lima, Peru) with the MiSeq Reagent Kit v3, generating paired-end reads (2 × 150 bp). Raw read quality was evaluated using FastQC v0.12.1, genome assembly was performed with SPAdes v4.2.0, assembly quality was assessed using QUAST v5.0.2, and genome annotation was conducted using Bakta v1.12.0.

Raw sequencing data were deposited in the NCBI Sequence Read Archive (SRA) under BioProject accession number PRJNA1431421.

### 4.21. Statistical Analysis

All experiments were performed in triplicate, and results are presented as mean ± standard deviation (SD). Statistical analyses were performed using GraphPad Prism version 8.0.1 (GraphPad Software, San Diego, CA, USA). Differences among groups were evaluated using one-way analysis of variance (ANOVA) followed by Tukey’s multiple-comparison test. Statistical significance was established at *p* < 0.05.

## 5. Conclusions

The integration of genome and metagenome mining, bacterial co-culture activation, and peptidomic analyses provided a complementary strategy for antimicrobial peptide discovery from South American environmental bacteria. Genome and metagenome mining enabled the in silico prioritization of antimicrobial peptide candidates and identified bacterial genera with promising biosynthetic potential for future investigation. Experimental analyses of bioactive secretomes obtained from environmental bacterial isolates demonstrated antimicrobial and antioxidant activities, while LC-MS/MS identified peptide sequences within active chromatographic fractions. Together, these findings support the use of genome mining and experimental evaluation of environmental bacterial secretomes as complementary strategies for antimicrobial molecule discovery.

## 6. Limitations of the Study

Although the present study identified promising antimicrobial peptide candidates through an integrated computational and experimental workflow, several limitations should be acknowledged. The antimicrobial activity was evaluated using bioactive peptide fractions rather than individual purified peptides, and therefore the contribution of each peptide sequence to the observed biological activity could not be established. Likewise, the peptide sequences identified by LC-MS/MS require further validation through peptide synthesis and functional assays. In addition, the biological activities predicted by the computational analyses, including anti-inflammatory and anticancer properties, were not experimentally evaluated. Finally, antimicrobial activity was assessed under in vitro conditions against a limited number of bacterial strains. Future studies should focus on validating the biological activity of individual peptides and evaluating their efficacy against a broader panel of clinically relevant pathogens.

## Data Availability

The datasets analyzed in this study are publicly available in the National Center for Biotechnology Information (NCBI). The genome and transcriptome data of *S. xiamenensis* LC6 are available under BioProject accession number PRJNA547647. Whole-genome sequencing data generated in this study have been deposited in the NCBI Sequence Read Archive (SRA) under BioProject accession number PRJNA1431421. The bioinformatic pipeline used for genome and metagenome mining of antimicrobial peptide candidates is available at: https://github.com/Abraham-Culupu/genome-metagenome-AMP-prediction (accessed on 10 February 2026). Additional data supporting the findings of this study are available from the corresponding author upon reasonable request.

## Supplementary Materials

The following supporting information can be downloaded at: https://www.mdpi.com/article/10.3390/antibiotics15070696/s1, Figure S1: Distribution of bacterial genera identified in South American genomic datasets containing predicted antimicrobial peptide (AMP) candidates. Donut charts show the relative abundance of bacterial genera detected in each country after screening with the Macrel pipeline (AMP probability score ≥ 0.6). Figure S2: Distribution of environmental sources represented in South American metagenomic datasets containing predicted AMP candidates. Donut charts show the relative proportions of environmental matrices from each country included in this study. Table S1: Environmental bacterial genomes from South America included in this study. Table S2: Environmental bacterial metagenomes from South America included in this study. Table S3: Predicted properties of genome derived antimicrobial peptides. Table S4: Predicted properties of metagenome derived antimicrobial peptides. Table S5. Biosynthetic gene clusters predicted using antiSMASH 8.0.
