# Supplementary figures and images for: Integrated Genome Mining, Bacterial Co-Culture Activation, and Peptidomic Analyses Identify Antimicrobial Peptide Candidates from South American Bacteria
Source: Antibiotics (Basel). 2026 Jul 16;15(7):696. doi: 10.3390/antibiotics15070696 (PMC13403791; doi:10.3390/antibiotics15070696)

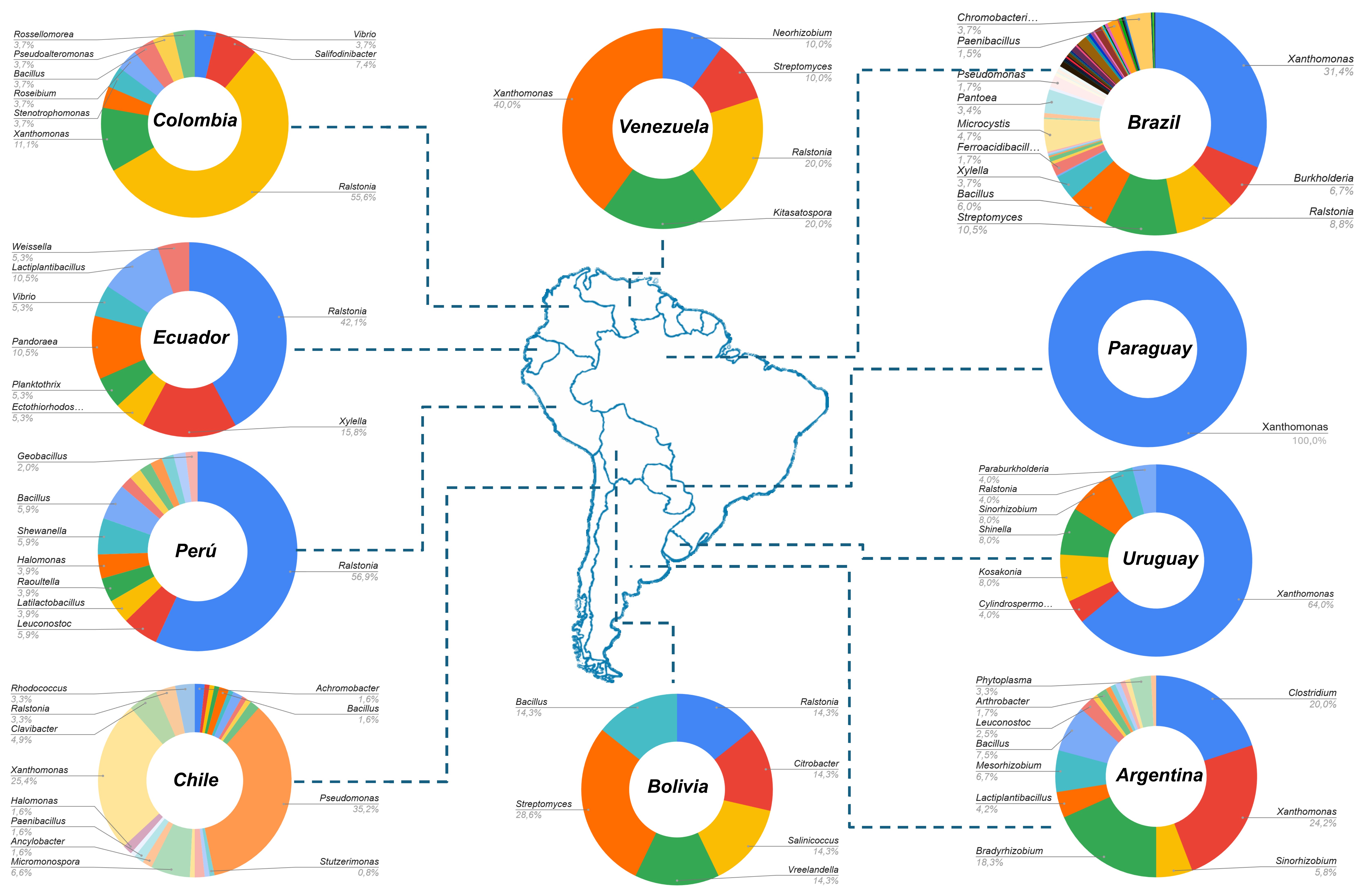

Supplement: Supplementary file 1 [file antibiotics-15-00696-s001.zip › Supplementary File/S_Figure 1.jpg]

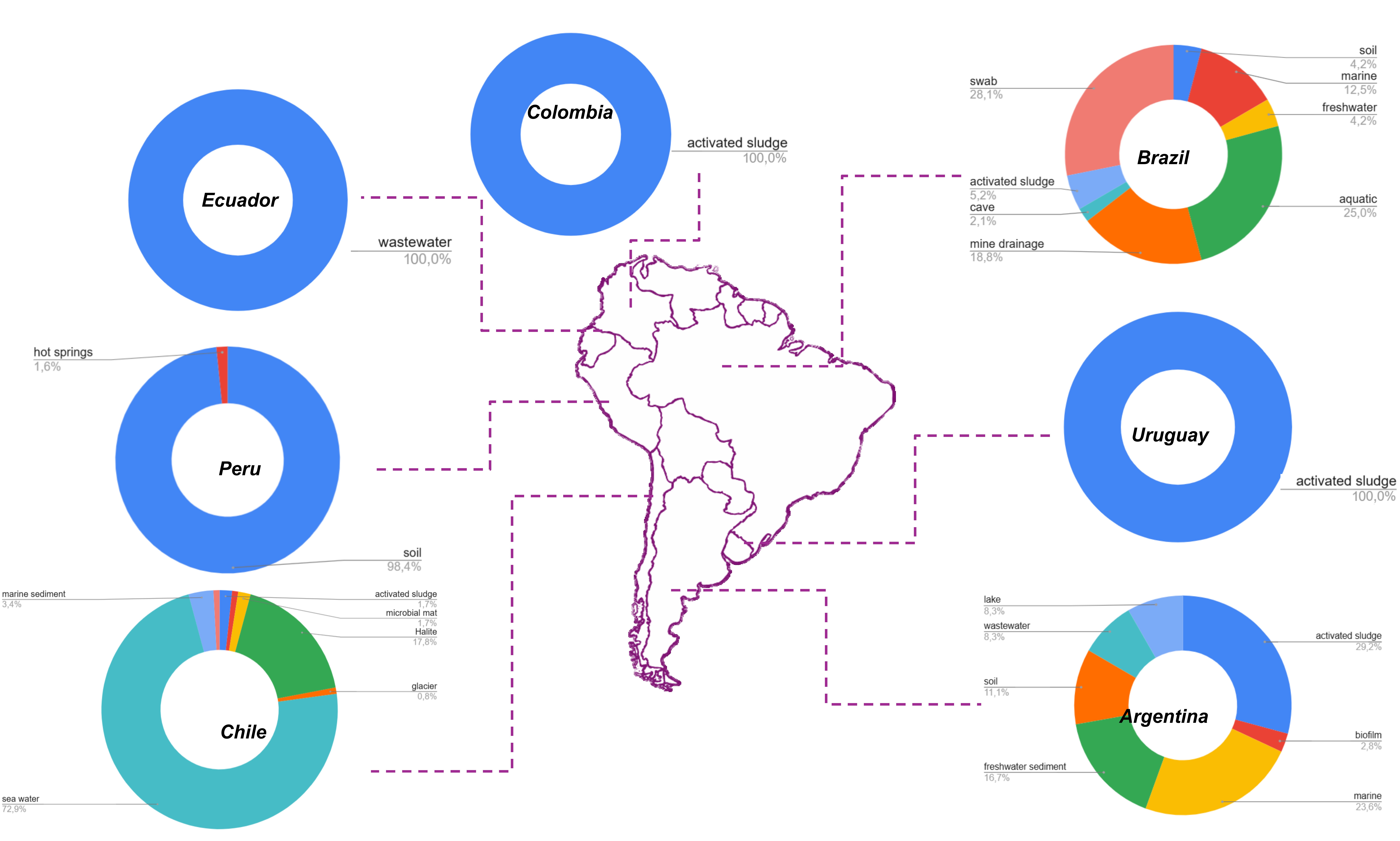

Supplement: Supplementary file 1 [file antibiotics-15-00696-s001.zip › Supplementary File/S_Figure 2.jpg]
